# Study of Imidazolium Salt Derivatives as PIK3CA Inhibitors Using a Comprehensive in Silico Method

**DOI:** 10.3390/ijms19030896

**Published:** 2018-03-18

**Authors:** Ming-yang Wang, Jing-wei Liang, Xin-yang Li, Kamara Mohamed Olounfeh, Shi-long Li, Shan Wang, Lin Wang, Fan-hao Meng

**Affiliations:** School of Pharmacy, China Medical University, Shenyang 110122, China; wmy940623@163.com (M.-y.W.); jwliang@cmu.edu.cn (J.-w.L.); xinyanglicmu@163.com (X.-y.L.); mohamedkamara6994@yahoo.com (K.M.O.); 13322489192@163.com (S.-l.L.); 18341653600@163.com (S.W.); lynnwang928@gmail.com (L.W.)

**Keywords:** QSAR, docking, network analysis, PIK3CA, molecular dynamic

## Abstract

A series of imidazolium salt derivatives have demonstrated potent antitumor activity in prior research. A comprehensive in silicon method was carried out to identify the putative protein target and detailed structure-activity relationship of the compounds. The Topomer CoMFA and CoMSIA techniques were implemented during the investigation to obtain the relationship between the properties of the substituent group and the contour map of around 77 compounds; the Topomer CoMFA and CoMSIA models were reliable with the statistical data. The protein–protein interaction network was constructed by combining the Pharmmapper platform and STRING database. After generating the sub-network, the phosphatidylinositol-4,5-bisphosphate 3-kinase catalytic subunit α (PIK3CA with protein data bank ID: 3ZIM) was selected as the putative target of imidazolium salt derivatives. A docking study was carried out to correlate interactions of amino acids in protein active pockets surrounded by the ligand with contour maps generated by the structure-activity relationship method. Then the molecular dynamics simulations demonstrated that the imidazolium salt derivatives have potent binding capacity and stability to receptor 3ZIM, and the two ligand-receptor complex was stable in the last 2 ns. Finally, the ligand-based structure-activity relationship and receptor-based docking were combined together to identify the structural requirement of the imidazolium salt derivatives, which will be used to design and synthesize the novel PIK3CA inhibitors.

## 1. Introduction

Cancer is the second leading cause of death, globally, and was responsible for 8.8 million deaths in 2015. Globally, nearly 1 in 6 deaths are due to cancer [1]. Chemotherapy is generally the main treatment for various cancers. Chemotherapeutic agents (anti-cancer drugs) have a range of side-effects such as immunosuppression, myelosuppression, anemia, teratogenicity, infertility, and even secondary neoplasm [2,3,4,5,6]. The major goal of oncology scientists is to design a selective and effective anticancer agent that is only sensitive in normal cancer cells, as well as the ability to predict, alter, or block the hallmark of cancer cells and is likely to improve the therapeutic index [7]. Therefore, the search for a targeted, effective drug with minimum toxicity is urgently necessary [3,7].

Previous studies have shown that the phosphatidylinositol-3-kinase (PI3K) signaling pathway is a crucial one for many aspects of cell growth and survival. Abnormalities in the PI3K pathway are common in cancer and have a role to play in neoplastic transformation [8]. The most frequent genetic aberrations in cancer are linked to somatic missense mutations in the gene encoding PIK3CA (p110α) [9]. Given the important role of the PI3K signaling pathway, some selective inhibitors—PX-866 and PEG Wortmannin—have entered into preclinical status [10,11].

Imidazolium salts serve as the nuclear skeleton in many compounds with anticancer activity [12,13,14], and some of them showed an inhibited effect of PI3K [15,16,17]. A series of imidazolium salt derivatives were designed and synthesized by molecular hybridization tools in the prior research, with the hybrid compound demonstrating potent cytotoxic activity against HL-60, A549 and MCF-7 tumor cell lines (the 77 hybrid compounds with the mean IC_50_ values of 2.84 μM) [18,19]. There was no further structure-function relationship, target or mechanism with respect to these novel imidazolium salt derivatives.

Structural modification of a familiar natural product, active compound or clinical drug is an efficient method for designing a novel drug. The main purpose of structural modification is to reduce the toxicity of target compound, while enhancing the utility of the drug [20]. This is generally done by altering the key substituent group in the nuclear skeleton of target compounds to increase the binding affinity and specificity to the active site of receptor protein, and improve ADME (absorption, distribution, metabolic and excretion), and changing the lipid-aqueous partition [20,21]. The most important step in drug design is to predict the target of a given compound and investigate the binding affinity for and specificity to the active target, which is achievable through the application of Computer-Aided Drug Design (CADD) techniques, which can improve the efficiency of this process [22].

Target identification is a fundamental step in the drug design pipeline and process, and makes use of PharmMapper. PharmMapper is a freely accessible web-based tool that is utilized for predicting the potential drug targets via a “reverse pharmacophore” (also known as “target fishing”) mapping method [23]. Benefiting from a highly efficient and robust mapping method, PharmMapper, with its high-throughput ability, is able to identify the potential target candidates from the database with a runtime of a few hours [23].

Protein–protein interactions (PPIs) can illustrate the interaction between two or more protein molecules that share a substrate in a metabolic pathway, regulate each other transcriptionally, or participate in larger multi-protein assemblies, under the PPI network [24]. Cancer-related proteins obtained by reverse docking techniques using the PharmMapper platform and the STRING database will be combined together to construct the PPI network. The weight of a node in the PPI network was determined by its own properties and its associated edges [25]; three centrality measures calculated by CytoNCA plugins Subgraph Centrality, Betweenness Centrality and Closeness Centrality [26,27,28] were utilized to generate the sub-network and to screen the potential target of the imidazolium salt derivatives.

The 3D-QSAR (three-dimension-quantitative structure-activity relationship) and docking techniques were regarded as effective and useful tools for drug discovery, a combined method consisting of ligand-based 3D-QSAR and receptor-based docking was utilized to identify the structural requirements of the imidazolium salt derivatives. A molecular dynamics (MD) simulation was utilized to estimate the strength of the intermolecular interaction between the imidazolium salt derivatives and their putative target. This integrated in silicon study not only limits these imidazolium salt derivatives, but could also serve as a guideline for identifying the target of other derivatives with potent activity and to modify the structure of these compounds based on the structure-activity relationship information obtained from the 3D-QSAR and docking study.

## 2. Results and Discussion

**Figure 1:**
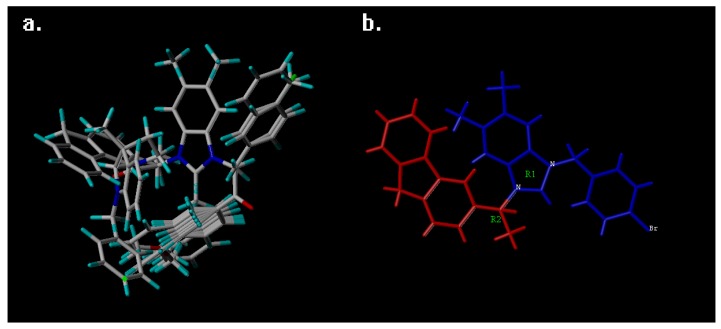
The result of the database alignment using CoMSIA technique (**a**) and topomer fragment method using the topomer CoMFA technique (**b**), the fragment 1 was painted in blue and the fragment 2 was painted in red.

After executing the fragment method in Figure 1a, the topomer CoMFA gave the *q*^2^ values of 0.648 and *r*^2^ values of 0.896, with 6 optimum components. The database results of CoMSIA alignment is shown in Figure 1b; all 77 compounds were aligned based on the template and common moiety, which also provided reliable statistical values: *q*^2^ of 0.714, *r*^2^ of 0.925, with optimum components of 7. Other statistical values, such as SEE, MAE, F-test value and predictive *r*^2^ value are shown in Table 1.

**Figure 2:**
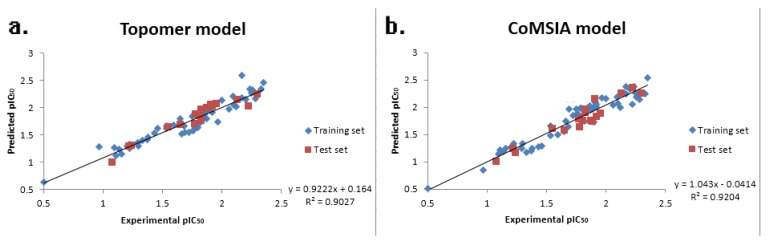
The experimental pIC_50_ values and predicted pIC_50_ values of the topomer CoMFA model (**a**) and the CoMSIA model (**b**).

The correlation coefficient (*R*^2^) between the Experimental pIC_50_ and predicted pIC_50_ of all 77 compounds is shown in Figure 2; the correlation coefficients of the topomer CoMFA and CoMSIA models were found to be 0.9027 and 0.9204, respectively, showing that the topomer CoMFA and CoMSIA models were reliable and precise for the prediction of activity.

**Figure 3:**
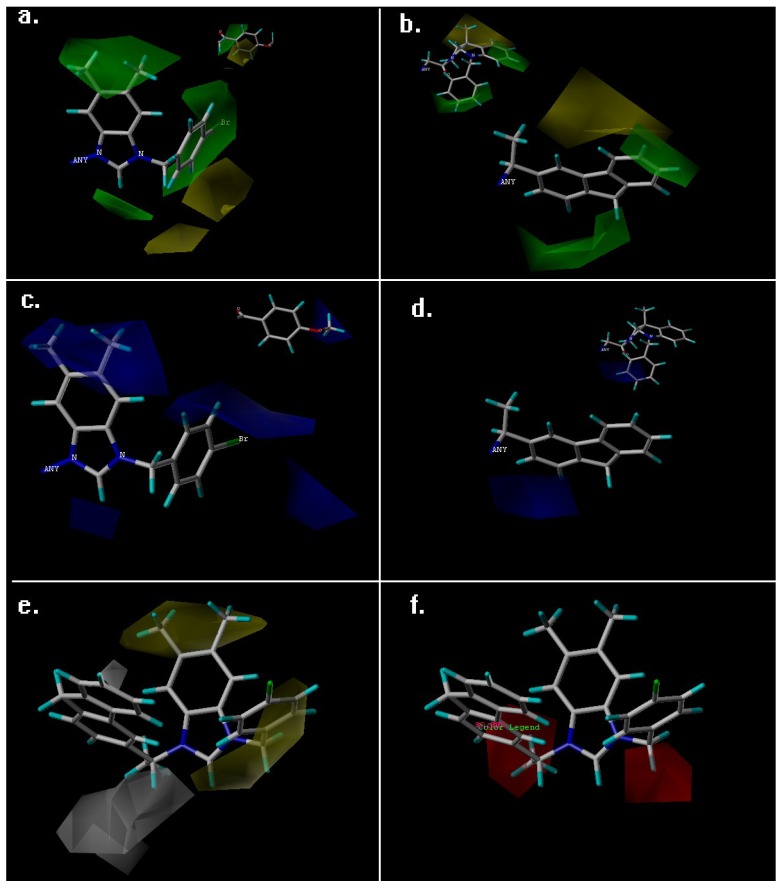
Contour maps of the results of topomer CoMFA and CoMSIA. (**a**,**b**) Topomer CoMFA steric contour maps of fragment 1 and 2, the green and yellow regions indicate the sterically favorable and unfavorable regions, respectively; (**c**,**d**) Topomer CoMFA electrostatic contour map of fragment 1 and 2, the blue and red regions are favorable to positively and negatively charged substituents, respectively; (**e**) CoMSIA hydrophobic contour map, the yellow and white regions are favorable and unfavorable to hydrophobic substituent groups, respectively; (**f**) CoMFA hydrogen bond acceptor field, magenta and red indicate regions favorable and unfavorable to hydrogen bond acceptor (HBA) atoms, respectively.

The steric and electrostatic contour maps of topomer CoMFA are shown in Figure 3a–d, and the hydrophobic and hydrogen-bond acceptor contour maps of CoMSIA are shown in Figure 3e,f. The structure of the most active compound—compound 72—was selected as the reference structure for the generation and visualization of the topomer CoMFA and CoMSIA contour maps.

The steric contour maps of the results of topomer CoMFA for fragment 1 and fragment 2 are shown in Figure 3a,b, respectively. In Figure 3a, the green contour map around the R1 substituent of imidazole/triazole ring indicates that this region was favorable for bulky groups. The results can be proved by the fact that compounds 72, 73 and 74 (with IC_50_ values of 0.45, 0.68, and 0.58 μM, respectively) with 5,6-Dimethyl-benzimidazole exhibit more potent cytotoxic activities than compound 47 and 45 (with IC_50_ values of 1.75 and 2.17 μM) without substituents in the imidazole ring. The green region was also near the R3 substituent; hence, the compounds with methyl in R2 shown (such as compound 14 with IC_50_ values of 4.18 μM and compound 07 with IC_50_ values of 5.94 μM) will increase their cytotoxic activities. 

The configuration change of the substituent group in R2 occurred when the hydroxyl in this place (in the upper right corner of Figure 3a) and the yellow region neared the substituent groups directly connected to the hydroxyl, which revealed that this place was sterically unfavorable for functional groups; for example, compounds 40 and 51, with 4-Bromobenzyl (with IC_50_ values of 1.73 and 1.09 μM, respectively) in the R2 substituent group, were more positively charged, resulting in more potent activity than compounds 36 and 53 with 4-Bromophenacyl (with IC_50_ values of 7.31 and 1.9 μM, respectively). 

Figure 3b shows the steric contour map of fragment 2, which is composed of two entirely different types of tricyclic substituent groups: flexible hexahydropyrrolo[2,3-b]indole substituents and rigid 9H-fluorene substituents. The green and yellow regions above the tricyclic substituent groups neared the 9H-fluorene and hexahydropyrrolo[2,3-b]indole, respectively; the combined region indicates that the compounds with 9H-fluorene substituents (35 compounds with mean IC_50_ values of 2.58 μM) demonstrated more potent activity than compounds with hexahydropyrrolo[2,3-b]indole substituents (42 compounds with mean IC_50_ values of 3.06 μM). In the case of hexahydropyrrolo[2,3-b]indole, the green region neared the *N*-benzyl group, revealing that the *N*-position was favorable for bulky groups, so compounds 23 and 24, possessing the N-benzyl moiety (with IC_50_ values of 1.27 and 1.29 μM, respectively), demonstrated higher activity than compounds 40 and 41 without substituents in the N-position (with IC_50_ values of 1.73 and 1.52 μM, respectively).

The topomer CoMFA electrostatic contour map of fragment 1 and 2 are shown in Figure 3c,d, respectively, in Figure 3c, the blue regions are found around the R1 and R3 regions, demonstrating that these positions were favorable for the electropositive group. Compounds in which the electropositive 5,6-Dimethyl-benzimidazole and 2-Methyl-imidazole moieties were present (compounds 4 and 72, with IC_50_ values of 0.47 and 0.45 μM, respectively) will demonstrate higher cytotoxic activity than compounds with imidazole, triazole and benzimidazole (compounds 46, 57 and 30, with IC_50_ values 3.49, 2.80 and 8.29 μM, respectively). The blue region near and around the benzene ring revealed that, whether or not there was hydroxyl in R2, the electropositive group was favorable for the activity, hence the compounds with electropositive methoxy and naphthyl benzene ring groups substituent in the benzene ring demonstrated more potent cytotoxic activity than those with electronegative fluorine and bromine substituents. It can be proved that compounds 36 and 40, with 4-Bromophenacyl and 4-Bromobenzyl (IC_50_ = 7.31 and 1.73 μM) exhibited weaker cytotoxic activity than the compounds 37, 38 and 42, with 4-methoxyphenacyl, 2-naphthylacyl and 2-Naphthylmethyl (with IC_50_ values of 6.23, 1.6 and 1.35 μM, respectively). In addition, the more the electropositive group is replaced in the benzene ring, the more the activity will decrease. For example, compound 75 (with IC_50_ values of 1.78 μM) with fluorine substituent in the benzene ring demonstrated weaker activity than compound 74 (with IC_50_ values of IC_50_ = 0.58 μM) with bromine substituent. There is no distinct red region in the electrostatic contour map of fragment 1. In Figure 3d, the blue region near the *N*-benzyl group indicates that this place is favorable for electropositive groups, and compounds with benzyl groups demonstrated more potent activity than compounds without substituents in the *N*-benzyl group, which is consistent with the steric contour map of fragment 2.

The CoMSIA hydrophobic contour is shown in Figure 3e, the yellow region indicates that R1 was favorable for hydrophobic groups, which can be validated by the fact that the compounds with bicyclic Benzimidazole skeletons exhibited more potent activity than the monocyclic imidazole skeletons. The yellow region can also be seen in the benzyl at the R2 and R3 positions, so the more hydrophobic group naphthyl in R2 position and methyl in R3 position demonstrated higher cytotoxic activity (compound 71, with an IC_50_ value of 0.57 μM) than other compounds that did not have this moiety. The white region around hexahydropyrrolo[2,3-b]indole indicates that the introduction of this hydrophobic skeleton was unfavorable for the cytotoxic activity, which is in accordance with the steric contour map of fragment 2; substituent groups with weak hydrophobicity were more beneficial for the activity than hydrophobic groups.

The CoMSIA hydrogen bond acceptor contour map is shown in Figure 3f, the red regions near the two sites of imidazole indicate that the introduction of the hydrogen bond acceptor carbonyl in this position was unfavorable for cytotoxic activity, which corresponds with the results of the topomer CoMFA steric contour map. There was no distinct magenta region around the compound, because the structural modifications of the hydrogen bond acceptors of compounds did not exhibit good biological activity.

**Figure 4:**
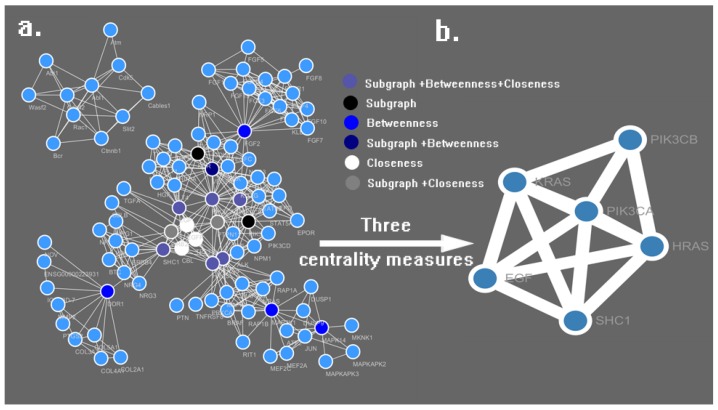
Network of cancer-related targets (**a**) and sub-network with essential targets (**b**), as generated by Cytoscape software and the CytoNCA plugin.

We received 3000 protein targets for 10 active compounds from the PharmMapper result list. After removing duplicates, 722 targets were used for screening cancer-related proteins; only proteins with clear cancer drug ligands can be used for further PPI analysis. Finally, 27 cancer-related proteins were identified, as shown in Table 2. Then all the proteins were uploaded to the STRING database to find their direct and functional partners and to obtain the primary PPI network of each protein. The software Cytoscape 3.5.0 (U.S. National Institute of General Medical Sciences, Bethesda, MD, USA) was utilized to merge the PPI network and analyze the merged network using its functional plugins. Finally, a network with 104 nodes and 496 edges was obtained, as shown in Figure 4a.

**Figure 5:**
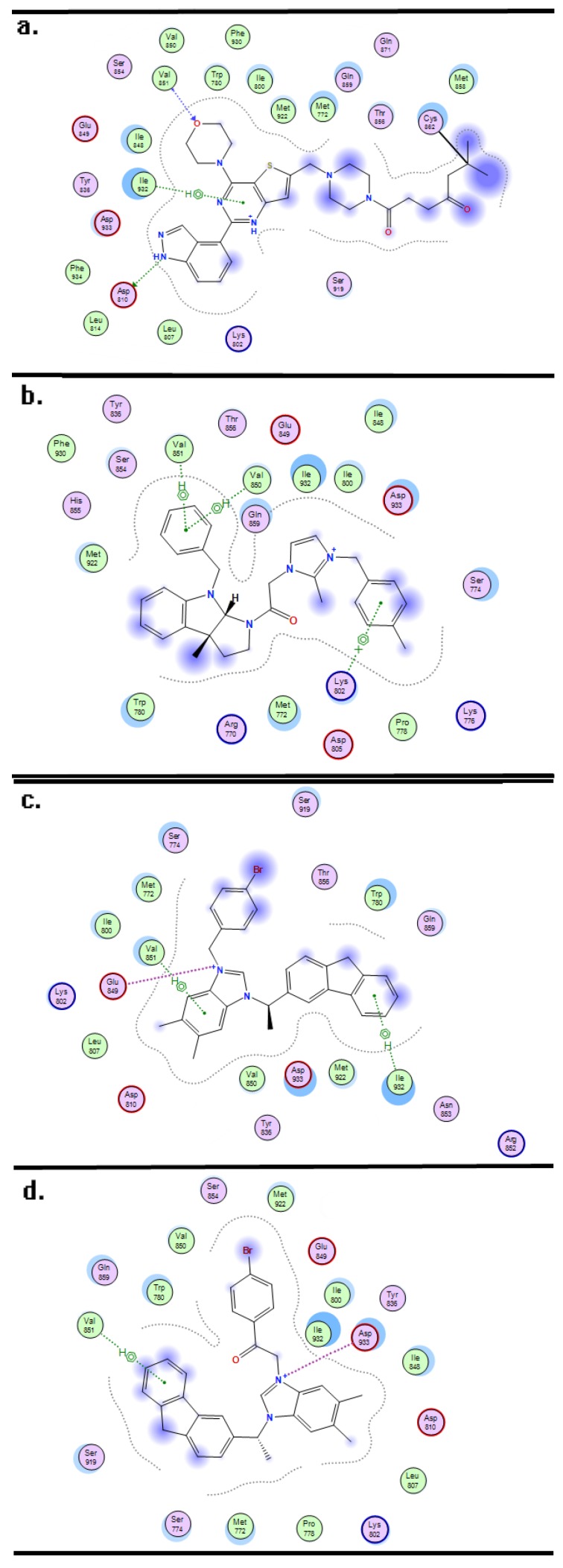
Molecular Dock interaction of CNX-1351 (**a**) and compounds 04 (**b**), 72 (**c**) and 74 (**d**) with the amino acids in the active pocket of protein 3zim.

The CytoNCA plugin was utilized to calculate the Subgraph Centrality, Betweenness Centrality and Closeness Centrality of all 104 nodes. After calculating the eight centrality measures of all nodes (see Table S1), all nodes were sorted by three centrality measures in descending order, the top 10% of the three centrality measures are colored black, blue and white respectively, and the overlapping nodes are colored with color mixtures, with the colored network being shown in the Figure. The top 10% of the three centrality measures were merged together to generate the sub-network with essential nodes, as shown in Figure 5b; in the merged list (see Table 3), the nodes are sorted by the three comprehensive centrality measures in descending order. The PIK3CA had the highest values for the three centrality measures, which indicates that PIK3CA produces some interactions with other proteins, and serves as a more essential node than the other proteins obtained from PharmMapper, hence the PIK3CA (with PDBID: 3ZIM in the PharmMapper results) was selected for the further docking study.

The 10 compounds had fit scores of 9.14 (compound 04), 7.77 (compound 54), 7.96 (compound 64), 8.25 (compound 66), 7.70 (compound 67) 7.99 (compound 70), 8.32 (compound 71), 8.52 (compound 72), 8.91 (compound 74) and 8.46 (compound 76) (see Table S2). The ligand interaction of compound 04, 72 and 74 is exhibited in Figure 5b–d, analyzing the interaction between the substituents in the compound skeleton and the key amino acids in the active pocket. 

Figure 5a exhibits the combinations of selective targeted covalent inhibitor CNX-1351 and PIK3CA, this ligand-receptor interaction was selected as the reference for analyzing the docking results of the three compounds, because the ligand-binding affinity to the receptor has been verified by in vitro biological experiment [29].

In Figure 5a, the inhibitor CNX-1351 formed several important interactions with amino acids Asp810, Val851, and Ile932. Similarly, in the three docking compounds, the Val851 formed arene–H interactions with the benzyl in *N*-benzyl group; this docking result was consistent with the steric, electrostatic and hydrophobic contour maps of topomer CoMFA and CoMSIA, which revealed that the benzyl in this position was beneficial for the improvement of the activity. In compounds 72 and 74, the Val 851 and Ile932 formed arene–H interactions with the 9H-fluorene moiety of the compound skeletons, while the hexahydropyrrolo[2,3-b]indole moiety of compound 04 did not form any interaction with the amino acid, indicating that compounds with rigid 9H-fluorene substituents were more similar to inhibitor CNX-1351 than compounds with flexible hexahydropyrrolo[2,3-b]indole rings; this also confirms the topomer CoMFA steric contour map and the CoMSIA hydrophobic contour map. In the docking results for compounds 04 and 72, the Val851 and Lys702 formed arene–H interactions with phenyl in the R2 and R1 positions, respectively, and as the result of CoMFA and CoMSIA, bulky, electropositive and hydrophobic groups were favorable for the cytotoxic activity. The imidazolium moieties of compounds 04, 72 and 74 formed ion contacts with acidic amino acids Asp933 and Glu849; the imidazolium served as the core moiety in a series of imidazolium salt derivatives. While performing structural modification of the imidazolium moieties of compounds 45, 46, 47, 49 and 50 (with IC_50_ values of 2.17, 3.49, 1.75, 1.10 and 1.01 μM, respectively), we found that the remodeled compounds 56, 57, 58, 59 and 60, which possess the triazolium moiety (with IC_50_ values of 2.05, 8.29, 2.07, 2.55 and 1.70 μM, respectively) did not exhibit any improvement in cytotoxic activity. Additionally, some greasy amino acids, basic amino acids and acidic amino acids around compounds 04, 72 and 74 were also similar to the inhibitor CNX-1351.

**Figure 6:**
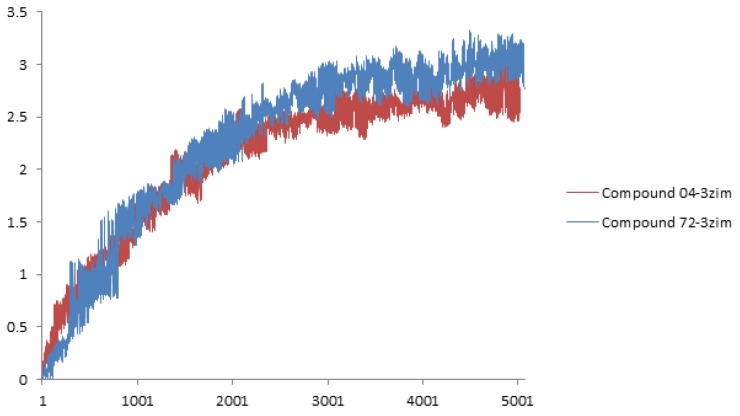
RMSD tendency of the ligand-receptor complex at different MD simulation times, red and blue curves indicate the compounds 04-3zim and 72-3zim complexes, respectively.

The molecular dynamics simulation results for the 04-3zim complex and the 72-3zim complex at different temperatures is shown in Figure 6. The values of root mean square deviations (RMSD) indicate that the transformation of the ligand-protein complex backbone forms the initial structure. In results of the compound 04-3zim, the RMSD values increased to 2.2 Å in 2.3 ns, and then maintained at between 2.5–3 Å until 5 ns; similarly, in the 72-3zim complex, the RMSD increased to 2.5 Å in 2.3 ns and then maintained at between 2.6–3.2 Å until 5 ns. The mean RMSD values of the 04-3zim complex and the 72-3zim complex were 2.115 and 2.253, respectively. The MD results revealed that these two active compounds had potent binding capacity and stability to the putative receptor. 

**Figure 7:**
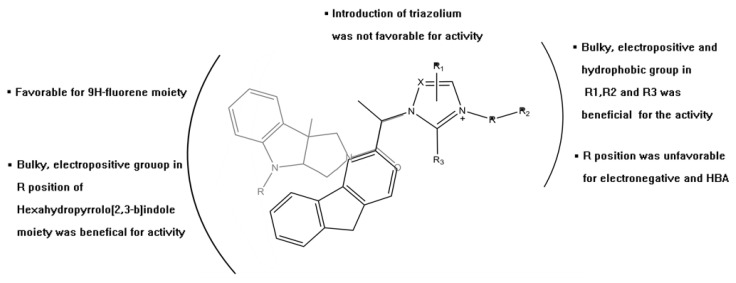
Structural requirements of a series of imidazolium salt derivatives demonstrate cytotoxic activity against tumor cells.

Finally, the structural requirement of a series of imidazolium salt derivatives was identified by a comprehensive method that combined ligand-based QSAR study and receptor-based docking-MD simulation (see Figure 7).

## 3. Materials and Methods 

### 3.1. QSAR Study

77 imidazolium salt derivatives were collected from two literature sources and summarized in Table 4, including 63 imidazolium salt derivatives and triazolium salt derivatives. The IC_50_ values of HL-60 tumor cell lines in the two literature sources were measured over a similar period of time and in the same laboratory [18,19], so we were able to merge these comparable IC_50_ values for the two series of compounds with common nuclear structures for further 3D-QSAR analysis. The IC_50_ values of all 77 compounds were converted to pIC_50_; these pIC_50_ values served as the dependent variable, while the descriptor values of CoMSIA and topomer CoMFA served as the dependent variables. The sketch function in software Sybyl X2.0 (Certara, Princeton, NJ, USA) was utilized to draw the structure of the compound, and these 77 molecules were divided in a ratio of 75:25 by Sybyl X2.0 in such a way that both datasets consisted of a balance of active and less-active molecules [30,31]. After minimizing the energy of the compound in the tripos force field with 0.05 kcal/(mol·A) termination and 1000 iterations, the compound was put into the database for topomer CoMFA and CoMSIA analysis in Sybyl X2.0 [32,33].

Topomer CoMFA included steric and electrostatic fields, while CoMSIA included steric, electrostatic, hydrophobic, hydrogen bond donor (HBD) atom, and hydrogen bond acceptor (HBA) atom fields. PLS (partial least squares) techniques associated these field descriptors with the activity value [30]. Many statistics, such as the values Leave One Out (LOO), optimal number of components (ONC), Standard Error of Estimation (SEE), mean absolute error (MAE), cross-validated coefficients (*q*^2^), and conventional coefficient (*r*^2^), were important in the evaluation of the 3D-QSAR model, and could be worked out using the PLS method. The QSAR model is said to be good when the *q*^2^ value is greater than 0.5 and the *r*^2^ value is greater than 0.6; because *q*^2^ and *r*^2^ values reflect the model soundness, the best QSAR model will have the highest *q*^2^ and r^2^ value, the lowest SEE, and optimal number of components [31]. In the case of topomer CoMFA analysis, the PLS leave-one-out (LOO) method with CoMFA standard options for variable scaling was implemented to investigate the topomer CoMFA model [32].

To build the topomer CoMFA QSAR model, the topomer technique was applied to split the molecules into two fragments (the cutting method is shown in Figure 4a) [32]. Database alignment was used to build the CoMSIA QSAR model [31,33]; the imidazole ring was identified as the common core moiety, and the most active compound—72—was selected as the template.

In the CoMSIA analysis, the values of ONC, SEE, and *q*^2^ were worked out by LOO validation, turning on USE SAMPLES, and components set to ONC. In the process of calculating *r*^2^, USE SAMPLES was turned off, and column filtering was set to 2.0 kcal·mol^−1^ to speed up the calculation without sacrificing information content [30,31,32,33], components were set to ONC, which was the optimal number of components calculated by performing a SAMPLES run. SEE and *r*^2^ were utilized to assess the non-cross-validated models.

### 3.2. PPI Network Construction and Analysis

PharmMapper serves as a valuable tool for identifying potential targets for novel synthetic compounds, newly isolated natural products, compounds with known biological activity, or existing drugs whose mechanism of action is unknown [23,34].

The top 10 compounds with the highest pIC_50_ values of all imidazolium salt derivatives were selected for subsequent study using PharmMapper, and the mol2 formats of these compounds were uploaded to the PharmMapper server, the parameters Generate Confomers and Maximum Generated Conformations were set to ON and 300, respectively, the target set Druggable Pharmacophore Models (v2017, 16159) served as pharmacophore mapping, and all other options conformed to the default settings [23,34].

Protein–protein interactions (PPIs) participate in many metabolic processes that occur in living organisms, such as cellular communication, immunological response, and gene expression control [24]. Systematic description of these interactions aids elucidation of the interrelationships among targets. Thus, targeting PPIs with small-molecule compounds is becoming an essential step to comprehending even major-target mechanisms [24]. Cancer-related targets of imidazolium salt derivatives were gathered by screening the results of PharmMapper, these proteins were used as the input protein in the STRING (https://string-db.org/) database [35] to search for related proteins or pathways that had previously been reported. Finally, we merged all the PPIs of cancer-related targets of imidazolium salt derivatives into an integrated network to identify the critical protein in Cytoscape 3.5.0 software.

Cytoscape is a free open-source Java application for visualizing molecular networks and integrating gene-expression profiles [36]. Plugins are available for network and molecular profiling analyses, new layouts, additional file format support and connection with databases and searching in large networks [36,37].

CytoNCA is a plugin in Cytoscape that integrates calculation, evaluation and visualization analysis for multiple centrality measures. There are 8 centrality measures provided by CytoNCA: Betweenness, Closeness Centrality, Degree Centrality, Eigenvector Centrality, Local Average Connectivity-based Centrality, Network Centrality, Subgraph Centrality, Information Centrality [38]. The centrality measurement analysis was conducted in order to identify the essential proteins in an already-built PPI network.

### 3.3. Molecular Docking and Dynamics

MOE (Molecular Operating Environment) is a comprehensive Computer-Aided Drug Design (CADD) software program that incorporates the functions of QSAR, molecular docking, molecular dynamics, ADME (absorption, distribution, metabolism, and excretion) and homologous modeling; all these functions can be regarded as conducive instruments in the field of drug discovery and biochemistry. The molecular docking and dynamics technology were performed in MOE2016 software to detect the stability and affinity between the ligands and predictive targets [39,40].

In the case of the docking study, the pdb formats of essential proteins were downloaded from the RCSB Protein Data Bank (http://www.rcsb.org/pdb/home/home.do), which served as the receptors, while the mol2 formats for the top 10 compounds with highest pIC_50_ were obtained from Sybyl software and served as the ligands. All the ligands will be docked at the active site of receptors in the manner of Triangle Matcher placement and Induced Fit refinement; the five docking conformations are listed in descending order by London dG functional score in the results panel. Based on the London dG functional score, the interaction between the structure of the compounds and the key amino acids in the active pocket can be visualized using the Ligand interaction function [40].

Integrated MD simulations for the ligand-receptor complex were performed using the program dynamics simulations, and all complexes were generated from the docking results. MD simulations were implemented in three phases: preparation, restrictive MD simulations, and non-restrictive MD simulations. During the preparation phase, the minimization and equilibration of constrained complexes were performed in a 15 Å3-sized water box [41], and an Amber12 EHT force field file was applied for energy minimization and equilibration with Gasteiger-Huckel charges using Boltzmann initial velocity. Restrictive MD simulations were carried out after the preparation phase, while gradually raising the system temperature from 300 to 310 K, and maintaining pressure at 101.325 kPa within 100 ps [42,43]. Finally, a 5 ns MD non-restrictive MD simulation with a 2 fs timestep, was carried out to investigate the stability and affinity of the ligand-receptor complex by contrasting the root mean square deviations (RMSD) [41,43].

## 4. Conclusions

A novel comprehensive method was carried out to identify the target and structural requirements of imidazolium salt derivatives. Cancer-related protein PIK3CA was identified as the putative target for a series of imidazolium salt derivatives by the PharmMapper platform and PPI network. In the following QSAR and docking study, the important substituent groups related to the cytotoxic activity formed molecular interaction directly with the key amino acids around the active pocket; further MD simulations showed the docking conformation to have potent binding affinity and stability. After correlating these ligand-based and receptor-based results, we found that bulky, electropositive and hydrophobic groups were beneficial for cytotoxic activity, and that compounds with imidazolium and 9H-fluorene moieties exhibited more potent activity than those with hexahydropyrrolo[2,3-b]indole and imidazolium moieties; additionally, the HBA and electronegative atoms on the two sides of the imidazole ring were adverse to the activity. This comprehensive method not only serves as a guideline for designing and synthesizing a novel imidazolium salt derivative PIK3CA inhibitor, but also provides new ideas for the structural modification of known compounds.

## Figures and Tables

**Table 1:** The partial least squares (PLS) statistical parameters for the CoMFA and CoMSIA.

PLS Statistical Parameters	Topomer CoMFA	CoMSIA
*q*^2 a^	0.648	0.714
*r*^2 b^	0.896	0.925
ONC ^c^	6	7
SEE ^d^	0.107	0.094
F ^e^	-	255.417
MAE ^f^	0.085	0.111
*r*^pred2 g^	0.914	0.947
Fraction of Field contribution ^h^		
steric	0.691	0.264
Electrostatic	0.309	0.196
Hydrophobic	-	0.221
H-bond acceptor	-	0.319
H-bond donor	-	0

^a^ Cross-validated correlation coefficient; ^b^ Non-cross-validated correlation coefficient; ^c^ Optimum number of components; ^d^ Standard error of estimate; ^e^ F-test value; ^f^ Mean absolute error for test set compound ^g^ Predictive *r*^2^ value; ^h^ Field: steric, electrostatic, hydrophobic, hydrogen-bond acceptor and hydrogen-bond donor.

**Table 2:** The 27 putative cancer-related proteins obtained from the PharmMapper platform.

PDB ID	Gene Names
1UWH 1UWJ	*BRAF BRAF1 RAFB1*
3BBT	*ERBB4 HER4*
3GCS 3HEG	*MAPK14 CSBP CSBP1 CSBP2 CSPB1 MXI2 SAPK2A*
3ZOS	*DDR1 CAK EDDR1 NEP NTRK4 PTK3A RTK6 TRKE*
3WZD 3WZE 4ASD	*KDR FLK1 VEGFR2*
3ZIM	*PIK3CA*
4MKC	*ALK*
4UOI	*KIT SCFR*
4TYJ 4UXQ	*FGFR4 JTK2 TKF*
4VO4	*FGFR1 BFGFR CEK FGFBR FLG FLT2 HBGFR*
1M17 1XKK 2ITO 3UG2 4G5J 4G5P 4HJO 4I1Z 4I22 4WKQ	*EGFR ERBB ERBB1 HER1*
5FV1	*VEGFA VEGF*
5L2I	*CDK6 CDKN6*

**Table 3:** Subgraph Centrality, Betweenness Centrality and Closeness Centrality of the nodes in the Sub-network.

No.	Name	Subgraph	Betweenness	Closeness
1	PIK3CA	112,381.20	3161.31	0.0781
2	HRAS	43,006.22	1533.64	0.0772
3	KRAS	40,547.32	1445.97	0.0771
4	SHC1	18,803.34	2494.36	0.0766
5	EGF	42,816.57	474.41	0.0763
6	PTPN11	42,259.02	376.02	0.0762

**Table 4:**
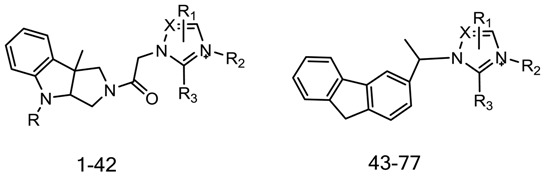
The structure of the 77 imidazolium salt derivatives with the IC_50_ values.

Compound	IC_50_	Imidazole/Triazole Ring	R_2_	R
1	7.75	2-Methyl-imidazole	4-Bromophenacyl	Bn
2	1.3	2-Methyl-imidazole	4-Methoxyphenacyl	Bn
3 *	2.2	2-Methyl-imidazole	4-Methylbenzyl	Bn
4	0.47	2-Methyl-imidazole	4-Methylbenzyl	Bn
5 *	5.72	Benzimidazole	Phenacyl	Bn
6	2.93	Benzimidazole	4-Bromophenacyl	Bn
7	5.94	Benzimidazole	4-Methoxyphenacyl	Bn
8 *	1.64	Benzimidazole	2-Naphthylacy	Bn
9	1.21	Benzimidazole	2-Bromobenzyl	Bn
10	1.42	Benzimidazole	4-Bromobenzyl	Bn
11 *	1.2	Benzimidazole	4-Methylbenzyl	Bn
12	4.64	2-Methyl-benzimidazole	Phenacyl	Bn
13	4.25	2-Methyl-benzimidazole	4-Bromophenacyl	Bn
14	4.18	2-Methyl-benzimidazole	4-Methoxyphenacyl	Bn
15 *	1.33	2-Methyl-benzimidazole	2-Bromobenzyl	Bn
16	1.35	2-Methyl-benzimidazole	4-Bromobenzyl	Bn
17	1.25	2-Methyl-benzimidazole	4-Methylbenzyl	Bn
18	1.38	2-Methyl-benzimidazole	2-Naphthylmethyl	Bn
19 *	1.47	5,6-Dimethyl-benzimidazole	Phenacyl	Bn
20 *	1.55	5,7-Dimethyl-benzimidazole	4-Bromophenacyl	Bn
21	1.64	5,8-Dimethyl-benzimidazole	4-Methoxyphenacyl	Bn
22	1.27	5,9-Dimethyl-benzimidazole	2-Bromobenzyl	Bn
23 *	1.29	5,10-Dimethyl-benzimidazole	4-Bromobenzyl	Bn
24	1.23	5,11-Dimethyl-benzimidazole	4-Methylbenzyl	Bn
25	1.21	5,12-Dimethyl-benzimidazole	2-Naphthylmethyl	Bn
26 *	5.98	Benzimidazole	2-Naphthylacyl	Me
27	5.07	Benzimidazole	2-Bromobenzyl	Me
28	6.96	Benzimidazole	4-Bromobenzyl	Me
29	5.13	Benzimidazole	4-Methylbenzyl	Me
30 *	2.8	Benzimidazole	2-Naphthylmethyl	Me
31	5.95	2-Methyl-benzimidazole	2-Bromobenzyl	Me
32	3.72	3-Methyl-benzimidazole	2-Bromobenzyl	Me
33	2.25	4-Methyl-benzimidazole	4-Methylbenzyl	Me
34 *	1.49	5-Methyl-benzimidazole	2-Naphthylmethyl	Me
35	8.03	5,6-Dimethyl-benzimidazole	Phenacyl	Me
36	7.31	5,6-Dimethyl-benzimidazole	4-Bromophenacyl	Me
37	6.23	5,6-Dimethyl-benzimidazole	4-Methoxyphenacyl	Me
38	1.6	5,6-Dimethyl-benzimidazole	2-Naphthylacyl	Me
39 *	1.67	5,6-Dimethyl-benzimidazole	2-Bromobenzyl	Me
40	1.73	5,6-Dimethyl-benzimidazole	4-Bromobenzyl	Me
41	1.52	5,6-Dimethyl-benzimidazole	4-Methylbenzyl	Me
42	1.35	5,6-Dimethyl-benzimidazole	2-Naphthylmethyl	Me
43	10.75	2-Methyl-benzimidazole	-	-
44	31.5	5,6-Dimethyl-benzimidazole	-	-
45	2.17	Imidazole	4-Bromobenzyl	-
46	3.49	Imidazole	Phenacyl	-
47	1.75	Imidazole	4-Bromophenacyl	-
48	2.92	Imidazole	4-Fluorophenacyl	-
49 *	1.1	Imidazole	4-Methoxyphenacyl	-
50	1.01	Imidazole	Naphthylacyl	-
51	1.09	2-Methyl-imidazole	4-Bromobenzyl	-
52 *	1.47	2-Methyl-imidazole	Phenacyl	-
53	1.9	2-Methyl-imidazole	4-Bromophenacyl	-
54	0.52	2-Methyl-imidazole	4-Methoxyphenacyl	-
55	0.79	2-Methyl-imidazole	Naphthylacyl	-
56	2.05	Triazole	4-Bromobenzyl	-
57 *	8.29	Triazole	Phenacyl	-
58	2.07	Triazole	4-Bromophenacyl	-
59	2.55	Triazole	4-Methoxyphenacyl	-
60	1.7	Triazole	Naphthylacyl	-
61 *	0.74	Benzimidazole	4-Bromobenzyl	-
62	0.76	Benzimidazole	Phenacyl	-
63	1.38	Benzimidazole	4-Bromophenacyl	-
64	0.56	Benzimidazole	4-Methoxyphenacyl	-
65 *	1.23	Benzimidazole	Naphthylacyl	-
66	0.6	2-Methyl-benzimidazole	4-Bromobenzyl	-
67	0.63	2-Methyl-benzimidazole	Phenacyl	-
68	0.81	2-Methyl-benzimidazole	4-Bromophenacyl	-
69	0.68	2-Methyl-benzimidazole	4-Fluorophenacyl	-
70 *	0.59	2-Methyl-benzimidazole	4-Methoxyphenacyl	-
71	0.57	2-Methyl-benzimidazole	Naphthylacyl	-
72	0.45	5,6-Dimethyl-benzimidazole	4-Bromobenzyl	-
73	0.68	5,6-Dimethyl-benzimidazole	Phenacyl	-
74	0.58	5,6-Dimethyl-benzimidazole	4-Bromophenacyl	-
75	1.78	5,6-Dimethyl-benzimidazole	4-Fluorophenacyl	-
76 *	0.5	5,6-Dimethyl-benzimidazole	4-Methoxyphenacyl	-
77	0.87	5,6-Dimethyl-benzimidazole	Naphthylacyl	-

* Text set.

## Supplementary Materials

Supplementary materials can be found at http://www.mdpi.com/1422-0067/19/3/896/s1.

## Abbreviations

QSAR	Quantitative Structure-Activity Relationship
CoMFA	Comparative of Molecular Field Analysis
CoMSIA	Comparative Molecular Similarity Indices Analysis
MD	Molecular Dynamics
